# Dual-Frequency Impedance Matching Network Design Using Genetic Algorithm for Power Ultrasound Transducer

**DOI:** 10.3390/mi15030344

**Published:** 2024-02-29

**Authors:** Wenchang Huang, Jiaqi Li, Shuai Wu, Yan He, Xiangxin Li, Zhitian Shen, Yaoyao Cui

**Affiliations:** 1 School of Biomedical Engineering (Suzhou), Division of Life Sciences and Medicine, University of Science and Technology of China, Suzhou 215163, China; hwc534@mail.ustc.edu.cn (W.H.); lijq@mail.ustc.edu.cn (J.L.); ws174060@mail.ustc.edu.cn (S.W.); heyan@mail.ustc.edu.cn (Y.H.); 18818272515@163.com (X.L.); 2 Suzhou Institute of Biomedical Engineering and Technology, Chinese Academy of Sciences, Suzhou 215163, China

**Keywords:** electrical impedance matching, power ultrasound transducer, dual-frequency ultrasound, ultrasound thermal ablation, genetic algorithm

## Abstract

Dual-frequency ultrasounds have demonstrated significant potential in augmenting thermal ablation efficiency for tumor treatment. Ensuring proper impedance matching between the dual-frequency transducer and the power amplifier system is imperative for equipment safety. This paper introduces a novel dual-frequency impedance matching network utilizing L-shaped topology and employing a genetic algorithm to compute component values. Implementation involved an adjustable capacitor and inductor network to achieve dual-frequency matching. Subsequently, the acoustic parameters of the dual-frequency HIFU transducer were evaluated before and after matching, and the effects of ultrasound thermal ablation with and without matching were compared. The proposed dual-frequency impedance matching system effectively reduced the standing wave ratio at the two resonance points while enhancing transmission efficiency. Thermal ablation experiments with matching circuits showed improved temperature rise efficiencies at both frequencies, resulting in an expanded ablation zone. The dual-frequency impedance matching method significantly enhances the transmission efficiency of the dual-frequency ultrasound system at two operational frequencies, thereby ensuring equipment safety. It holds promising prospects for application in dual-frequency ultrasound treatment.

## 1. Introduction

Ultrasounds are increasingly employed in the thermal ablation of tumors, owing to their minimal side effects and controllable energy distributions [1]. Leveraging the thermal effects of ultrasounds in tissues, hyperthermia can be effectively conducted, elevating the temperature of tumor tissue in the targeted area to surpass 65 °C. This induces deformation and necrosis in the tumor tissue, presenting a viable approach for treating tumors and vascular diseases [2]. The unique characteristics of ultrasound afford more precise energy delivery, high selectivity, minimal invasiveness, and substantial treatment volumes compared to alternative energy modalities utilized in hyperthermia surgery [3].

Due to the unique advantages of ultrasounds, ultrasound thermal ablation technology has received widespread attention in various fields of medical intervention and has achieved remarkable achievements [4,5]. Current high-intensity ultrasound can be divided into high-intensity focused ultrasound (HIFU) and catheter ultrasound (CBUS), each of which are designed for specific application scenarios [6,7].

High-intensity focused ultrasound, often called HIFU, uses the permeability and focusing properties of ultrasound to focus low-energy ultrasound waves from the outside onto the target site, creating an area of higher intensity. Notwithstanding the precision inherent in HIFU treatment, prevailing thermal ablation procedures entail external mechanical scanning, traversing from point to line until comprehensive coverage of the tumor area is attained. This protracted process extends the treatment duration to several hours, thereby diminishing treatment efficiency [8].

Serving as an alternative to extracorporeal HIFU treatment, catheter-based ultrasound leverages a diminutive ultrasound probe within a catheter to administer high-intensity ultrasound to the target site via natural orifices or intervention. It can be precisely positioned directly within or in proximity to the target body, necessitating fewer post-reach operations and affording a more condensed treatment duration [9]. However, the dimensions of interventional ultrasound instruments impose constraints, predominantly relying on a solitary transducer and exhibiting limited treatment precision.

In recent times, there has been extensive exploration into dual-frequency ultrasound transmission technology [10,11]. Dual-frequency ultrasound has the potential to augment the effectiveness of current treatments without necessitating intricate systems and probes, showcasing technical compatibility and applicability. Single-transducer dual-frequency ultrasound therapy exhibits conspicuous advantages over conventional single-frequency ultrasound. Dual-frequency ultrasound stimulation can enhance the acoustic cavitation effect more effectively than single-frequency ultrasound [12,13]. The incorporation of dual frequencies in HIFU not only augments treatment efficiency but also abbreviates the overall treatment duration [14]. Concurrently, dual-frequency ultrasound yields stable and larger lesions with reduced positional shifts [15]. In the realm of dual-frequency CBUS thermal ablation, there emerges an enhancement in treatment precision and control [16]. This advancement facilitates more meticulous interventional procedures, thereby refining the safety and efficacy of CBUS thermal ablation [17].

Nevertheless, piezoelectric materials employed in ultrasound thermal ablation applications exhibit a high response at resonant frequencies. Consequently, transducers fashioned from such materials are typically single-frequency and narrow-bandwidth, posing a substantial obstacle to the realization of dual-frequency ultrasound transmission from a single transducer.

While transducers commonly possess high-order resonant frequencies, the impedance values at these frequencies often deviate from the output impedance of the power amplifier system [18]. This impedance mismatch can result in diminished power transmission efficiency, prompting the need for electrical impedance matching (EIM) networks to avert issues arising from such disparities [19]. The conventional transducer matching primarily addresses a single frequency, accomplished through a basic LC EIM circuit with unique components [20]. Typical dual-frequency ultrasound transmission often mandates distinct matching circuits for each of the two frequencies [15]. Regrettably, these EIM networks fail to facilitate simultaneous ultrasound transmission at both frequencies, thereby curtailing their versatility. Ensuring the impedance values of the transducer at the two frequency points match the output impedance of the power amplifier system is crucial for the safety of dual-frequency ultrasound systems. A well-designed dual-frequency electrical impedance matching (DFEIM) network can match the impedance values of the transducer at two frequency points with the output impedance of the power amplifier system. This enables a single transducer to function at multiple resonant frequencies simultaneously, presenting a streamlined dual-frequency ultrasound transmission scheme. Nevertheless, there is a paucity of existing studies exploring DFEIM methods for power transducers.

Most research on EIM of ultrasound transducers has been focused on a single frequency [18,20]. Acoustic impedance matching enhances the narrow band of ultrasound imaging but tends to increase electrical impedance. Broadband matching for the transducer has the potential to improve the signal-to-noise ratio in ultrasound imaging [21]. Huang et al. proposed methods for achieving broadband matching [22]. An et al. introduced a broadband matching technology based on a genetic algorithm [23]. Given that the impedance can vary due to factors such as temperature and the surrounding medium during transducer operation, real-time impedance matching technology has garnered attention in research. Jin et al. proposed an online real-time matching system [24], while Gu et al. presented a novel adaptive matching method [25]. EIM has also been extensively studied in other domains, including antennas and electronic equipment operating at radio frequencies [26,27,28,29,30,31]. Lee et al. suggested a dual-frequency matching circuit using two parallel-connected matching circuits [32]. However, there is a relative scarcity of impedance studies specifically focused on dual-frequency power ultrasound.

This paper introduces an innovative method to implement a DFEIM network, with the objective of augmenting device safety and optimizing the efficiency of transmission in transducers and power amplifier systems. Firstly, the topology and selection method of the DFEIM network are proposed. Next, a genetic algorithm is utilized to calculate the values of the component parameters. Subsequently, the topology and parameter values of the DFEIM network are implemented in the adjustable DFEIM network and monitored through the feedback circuit. Finally, the dual-frequency transducer was tested with and without matching acoustic parameters and evaluated in thermal ablation experiments in a phantom.

## 2. Materials and Methods

### 2.1. DFEIM Principle

EIM seeks to match the input impedance of the transducer with the output impedance of the power amplifier, thereby mitigating power reflection and enhancing system safety. A typical method involves employing the Smith circle for designing the matching circuit [33]. In the case of the uncomplicated L-shaped EIM network, rearranging the placement sequence of the parallel inductor and series capacitor typically yields two distinct L-shaped topologies encompassing the entire Smith circle [24].

**Figure 1:**
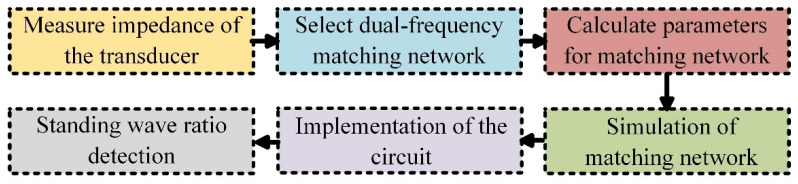
Design methodology for a DFEIM network.

The DFEIM matching process is delineated in Figure 1. Initially, a network analyzer (E5061B, Agilent Technologies, Inc., Santa Clara, CA, USA) was employed to evaluate the frequency response of the transducer. Next, the DFEIM topology was selected, and a genetic algorithm was utilized to determine the values of the circuit components. Subsequently, simulations were conducted with the selected topology and component values. The subsequent step entails the main control unit overseeing the actual implementation of the DFEIM network within the circuit. Finally, the feedback circuit is utilized to monitor the standing wave of the matched circuit.

**Figure 2:**
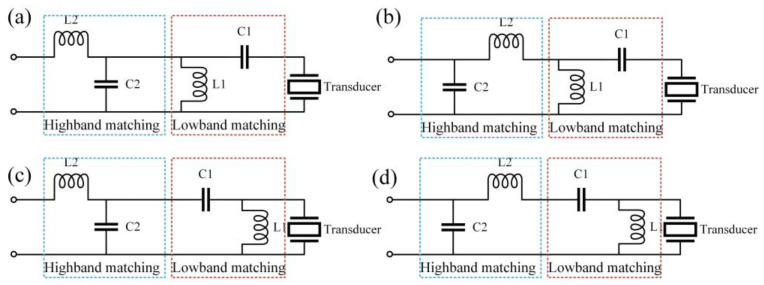
Four DFEIM network topologies derived from the L-shaped network paradigm: (**a**) the first type, (**b**) the second type, (**c**) the third type, and (**d**) the fourth type.

Diverging from the conventional approach of single-frequency EIM, the task of DFEIM necessitates consideration for two distinct frequency points. If these two frequency points undergo separate matching processes, the resulting networks may mutually interfere. Consequently, a holistic design for a DFEIM network is imperative. Drawing inspiration from the uncomplicated L-shaped matching network as a prototype, we fashioned a DFEIM network by consecutively linking two L-shaped EIM networks. To alleviate the mutual interference between the two frequency bands, the design decision was made: the L-shaped EIM network in the high-frequency band adopted a low-pass filter structure, while the L-shaped EIM network in the low-frequency band embraced a high-pass filter structure. The DFEIM network, as delineated in Figure 2, can be classified into four types based on the order of altering series and parallel capacitance and inductance, as illustrated.

**Figure 3:**
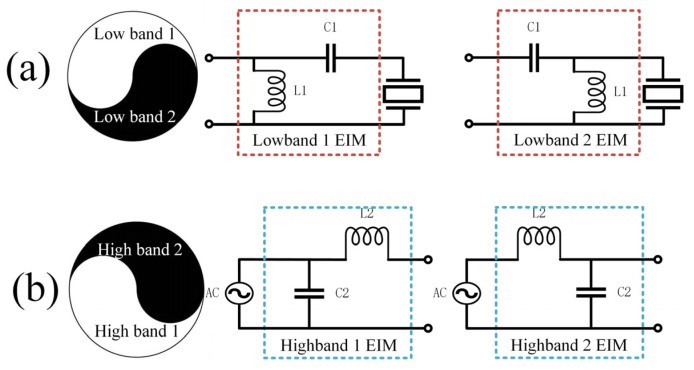
Methodology for choosing L-shaped topologies for (**a**) low-frequency band and (**b**) high-frequency band in DFEIM.

The choice of network type was primarily guided by the frequency response of the transducer at two distinct frequency points. As illustrated in Figure 3, when the low-frequency band occupies the upper left half of the Smith circle, the connection sequence involves initially connecting the capacitor followed by linking the inductor. Conversely, when the low-frequency band resides in the lower right half of the Smith circle, the initial connection entails the inductor, followed by the series connection of the capacitor. This configuration facilitates the establishment of a high-pass filter, thereby minimizing the impact on higher frequencies.

### 2.2. Genetic Algorithm

After determining the DFEIM network topology, it becomes crucial to determine the parameter values of its components. While single-frequency matching can compute component parameter values using the Smith circle method, DFEIM requires a broader search space for multiple parameters. The genetic algorithm mimics the principles of natural selection and inheritance, allowing for the simultaneous consideration of multiple parameters [34]. This approach enables a comprehensive exploration of the entire parameter space, ultimately leading to the identification of the global optimal solution or a solution closely approximating optimality.

In previous studies focusing on single-frequency EIM, genetic algorithms have demonstrated their ability to quickly identify suitable parameter combinations. However, there has been limited exploration of DFEIM in existing research. In this context, we apply a similar methodology, cleverly adapting it to the domain of DFEIM. With this innovative approach, we determine the parameter combinations for components in the DFEIM network.

**Figure 4:**
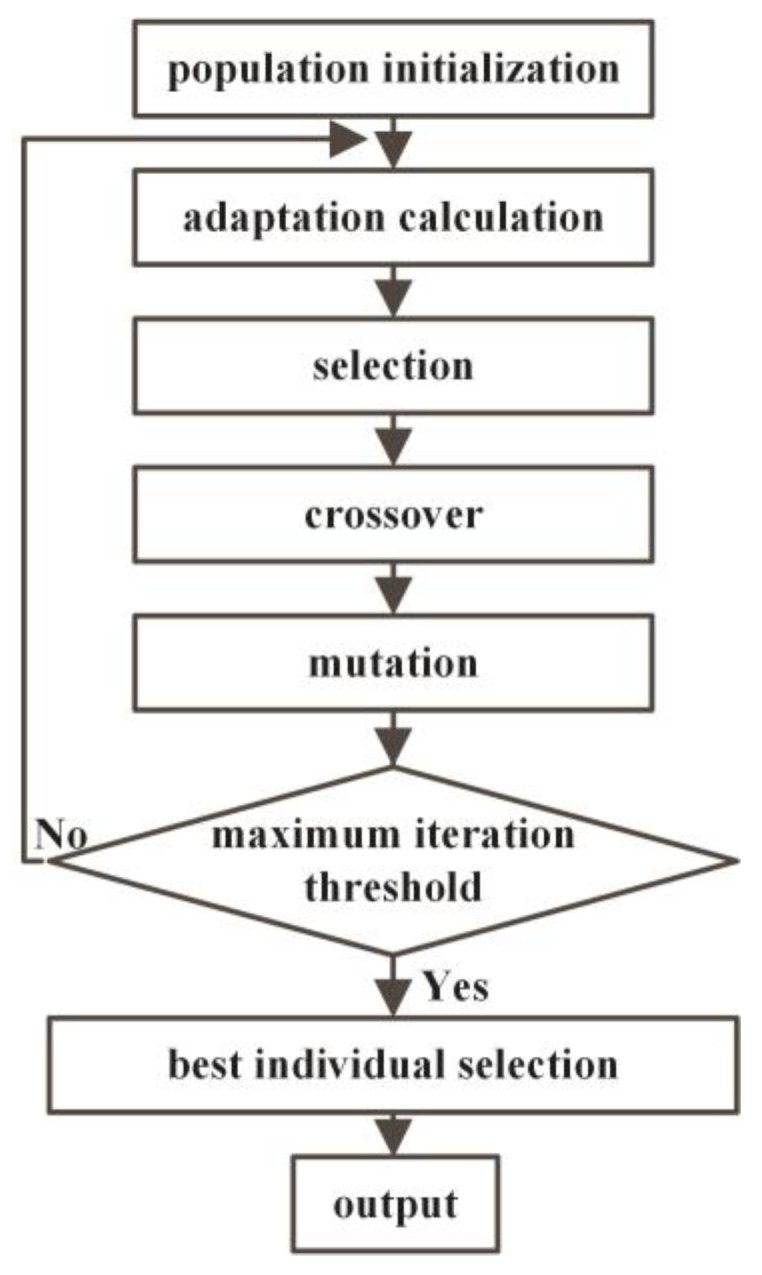
Genetic algorithm flow chart.

We performed an implementation of the genetic algorithm in MATLAB R2021a (MathWorks Inc., Natick, MA, USA). The genetic algorithm process is illustrated in Figure 4. Herein, the population limit range was defined, with inductance values ranging from 10 nH to 5000 nH and capacitance values spanning from 10 pF to 5000 pF. The population size was established at 90 individuals. The fitness function was articulated as the summation of the distances between impedance values at the two frequency points within the Smith circle and the target impedance value, as expressed in the subsequent equation:

fitness = abs (Z_f1_ − Z_0_) + abs (Z_f2_ − Z_0_), (1)

where Z_f1_ is the impedance value of frequency one, Z_f2_ is the impedance value of frequency two, and Z_0_ is the target impedance value. The output impedance of the power amplifier is selected here, and the zero-phase 50 Ohm impedance is used.

Through this approach, a parameter combination closely aligned with the target impedance values at the two frequency points can be derived. After genetic crossover and mutation, once the maximum genetic generation count of 500 is reached, the optimal parameter combination is output.

### 2.3. Adjustable DFEIM Network

**Figure 5:**
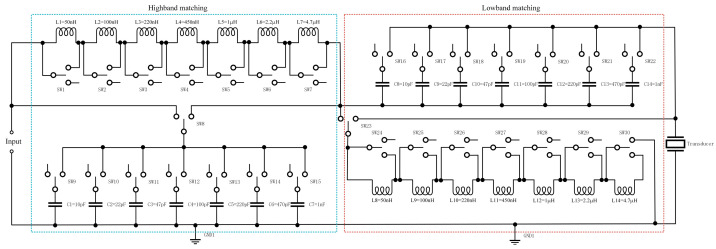
Schematic representation of a modifiable DFEIM system.

The configurable DFEIM network, as depicted in Figure 5, utilizes inductor and capacitor networks as adjustable components. The configuration of the DFEIM network, including the parameter values of capacitors and inductors, is controlled by relays. Relay 8 and relay 23 govern the configurations of L-shaped networks in the high-frequency and low-frequency bands, respectively.

Relays 1–7 regulate the inductance values of the series inductor in the high-frequency band, encompassing 50 nH, 100 nH, 220 nH, 450 nH, 1000 nH, 2200 nH, and 4700 nH. Relays 9–15 control the capacitance values of the parallel capacitor in the high-frequency band, ranging from 10 pF to 1000 pF. Additionally, relays 16–22 manipulate the capacitance values of the series capacitors in the low-frequency range (10 pF, 22 pF, 47 pF, 100 pF, 220 pF, 470 pF, and 1000 pF), while relays 24–30 adjust the inductance values of the parallel inductor in the low-frequency band (50 nH, 100 nH, 220 nH, 450 nH, 1000 nH, 2200 nH, and 4700 nH).

The selection of these parameter values in the adjustable DFEIM network is informed by empirical knowledge, as ultrasound thermal ablation typically occurs within the frequency range of 1 to 20 MHz, rendering this network versatile enough to accommodate a broad spectrum of application scenarios.

### 2.4. Design of the Feedback Circuit

**Figure 6:**
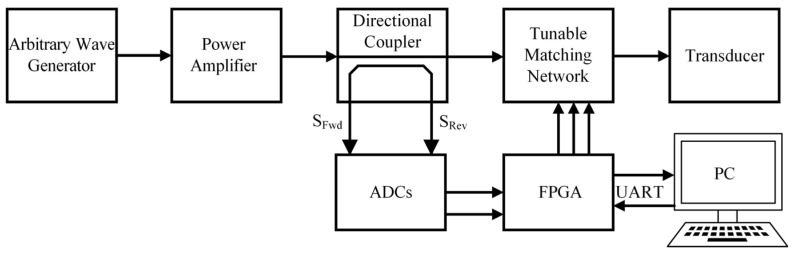
DFEIM system featuring a feedback circuit.

To establish a closed-loop monitoring system for assessing the effectiveness of the matching circuit, a DFEIM system, incorporating a feedback circuit as depicted in Figure 6, has been devised. The system consists of a signal generator, power amplifier, EIM network, and a feedback mechanism provided by a coupler [25]. Real-time collection of incident power and reflected power from the coupler is achieved by two ADCs (3PA1030, 3PEAK Inc., Suzhou, China) interfaced with the FPGA (Altera Cyclone Ⅳ, Intel Co., Ltd., Santa Clara, CA, USA), which subsequently transmits this data to the PC through the serial port.

### 2.5. Transducer Testing and Phantom Thermal Ablation Experiments

**Figure 7:**
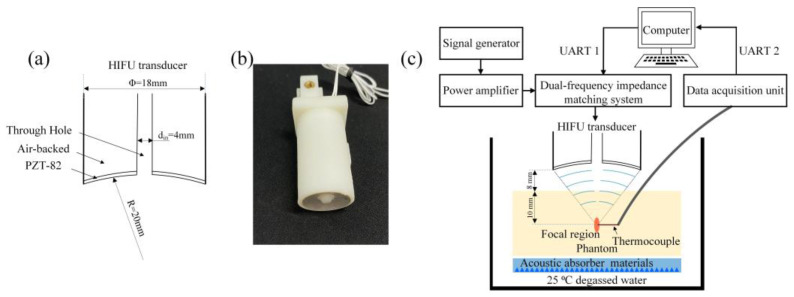
(**a**) The schematic of HIFU transducer, (**b**) photo of the HIFU transducer to be matched, (**c**) experiment setup for HIFU thermal ablation in the phantom.

The schematic of HIFU transducer depicted in Figure 7a represents an annular focused concave HIFU transducer utilized in our experiment. This customized transducer (Zibo Yuhai Electronic Ceramic Co., Ltd., Zibo, China) features an air backing and possesses a radius of curvature of 20 mm, a peripheral diameter of 18 mm, and a center hole diameter of 4 mm. The piezoelectric material employed is PZT-82. During the experiment, the transducer operated at its main resonant frequency of 4.6 MHz and third harmonic frequency of 13.8 MHz. Additionally, the fabricated HIFU transducer, illustrated in Figure 7b, serves as the object for EIM. Our strategy involves matching its impedance value at the primary resonant frequency and third resonant frequency with that of the power amplifier. A network analyzer was employed to examine the frequency response and standing wave ratio of the transducer without and with the DFEIM circuit. The sound power output was measured using a radiation force balance (RFB, Precision Acoustics Ltd., Dorchester, UK) under 18W electrical power conditions without and with the DFEIM circuit. Subsequently, an ultrasound thermal ablation experiment in the phantom was designed [35], as illustrated in Figure 7c. The phantom utilized in the thermal ablation experiments consisted of a polyacrylamide (PAA) gel infused with egg white, with the egg white concentration set at 40%. Aside from the whitened appearance of the thermal lesion area, the gel exhibits transparency, and its acoustic characteristics closely resemble those of soft tissue. High-frequency acoustic absorber materials (Aptflex F28, Precision Acoustics Ltd., Dorchester, UK) are placed beneath the prosthesis to absorb excess ultrasound energy. The thermal ablation lesion area at the two frequencies was compared without and with the DFEIM circuit. Employing a thermocouple (KPS-JY-T, Xinghua Suma Electric Instrument Co., Ltd., Xinghua, China), temperature data near the focal point were collected by a DAQ (Xunyan Electronic Co., Ltd., Yiwu, China). The focal point of the transducer is approximately 18 mm from its surface. We positioned the phantom roughly 8 mm away from the transducer surface, inserted a 0.2 mm diameter thermocouple into the focus of the phantom (10 mm from the phantom surface), and ensured alignment of the thermocouple’s temperature sensing area with the central symmetry axis of the transducer. In this setup, the thermocouple is situated near the focus of the transducer. Temperature readings from the thermocouples are captured twice per second by the DAQ and transferred to the computer for analysis. Finally, the thermal lesion areas in the phantom were observed and subjected to comparison.

## 3. Results

### 3.1. Topology of the Dual-Frequency DFEIM Network

**Figure 8:**
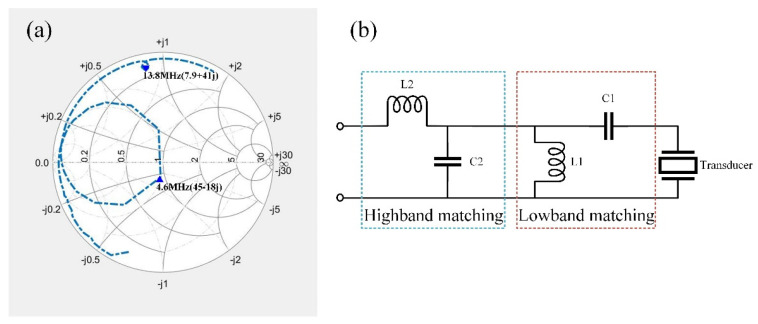
(**a**) The frequency response of the transducer without DFEIM, (**b**) the selected DFEIM topology, determined by the positions of the frequency response in the Smith circle.

Figure 8a depicts the frequency response of the transducer, measured with a network analyzer. The fundamental frequency registers at 4.6 MHz with an impedance value of 45 − 18j. The third harmonic frequency is observed at 13.8 MHz, exhibiting an impedance value of 7.9 + 41j. Figure 8b illustrates the chosen DFEIM network topology based on the positioning of the frequency response in the Smith circle.

### 3.2. The Parameter Values Acquired through the Genetic Algorithm and Implementation

**Figure 9:**
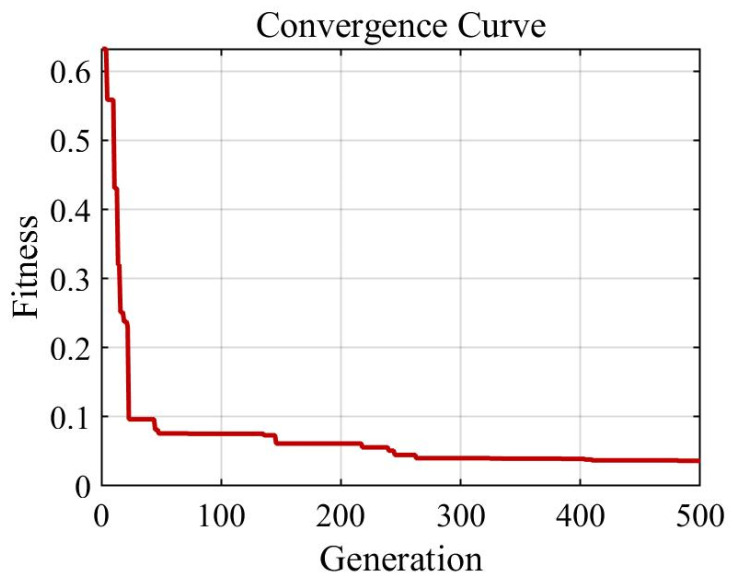
The correlation between the fitness function and the genetic algorithm.

The component values in the DFEIM network were computed through a genetic algorithm. After 500 iterations, the fitness function gradually converged, as depicted in Figure 9, illustrating the relationship between the fitness function and the genetic algorithm. The acquired component parameter values are outlined in Table 1.

**Figure 10:**
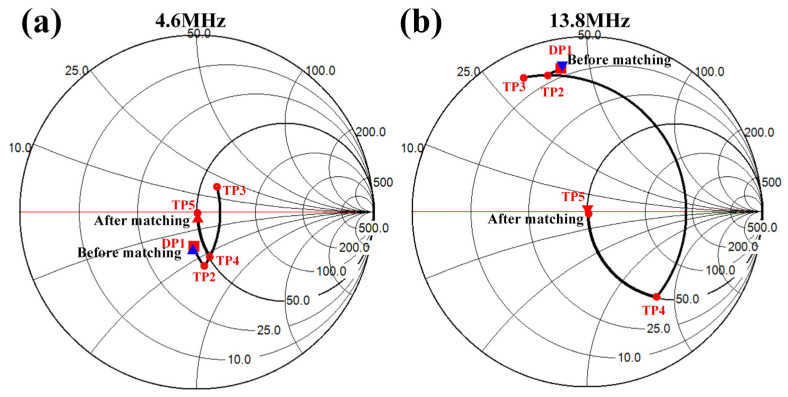
Theoretical verification of DFEIM network in Smith chart at (**a**) fundamental and (**b**) harmonic.

Subsequently, MATLAB R2021a transmitted the DFEIM network topology type and component parameter values to the FPGA via UART. The FPGA, in turn, controlled the corresponding IO port to implement the DFEIM circuit. In this process, relay 8 was engaged, and relay 23 was disengaged, resulting in a dual-frequency matching circuit topology as illustrated in Figure 8b. The activation of relays controlling capacitors and inductors facilitated the realization of adjustable parameter values of the components. Figure 10 shows the impedance changes at two frequencies. The impedance trajectory progresses from DP1 to TP2 to TP3 to TP4 to TP5. At 4.6 MHz, the impedance value changes from (45 − 18j) to (45 − 30j) to (60 + 18j) to (50 − 28j) to (50 − 1j), while, at 13.8 MHz, the impedance value changes from (7.9 + 41j) to (7.9 + 37j) to (5.6 + 31j) to (51 − 81j) to (51 − 2j). The simulation results indicated that the parameter values obtained by the genetic algorithm could simultaneously approximate the target impedance at two different frequency points.

### 3.3. Evaluation of the Feedback Circuit

To facilitate real-time monitoring of the matching process, the efficacy of the feedback circuit was assessed. This circuit is designed to capture voltage values representing the incident and reflected power, which are detected and converted by an ADC. A commercial power meter (RS-70, Taiwan Nissei Sokki Co., Ltd., New Taipei, China) was employed for comparative analysis.

**Figure 11:**
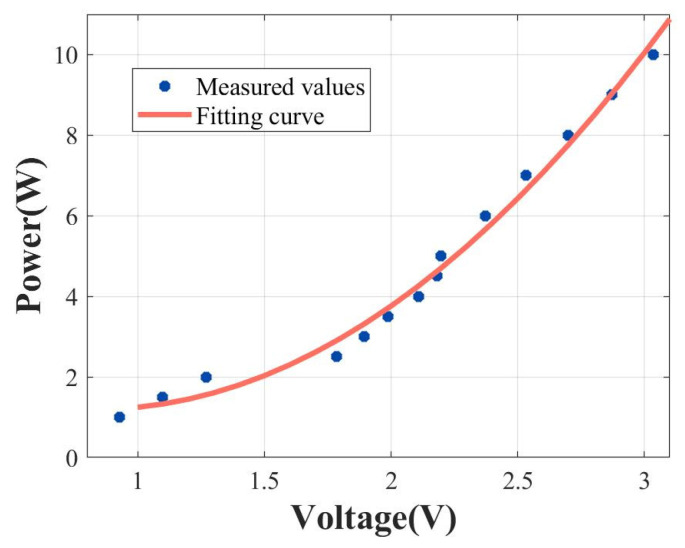
The correlation between the measured voltage and actual power within the feedback circuit, along with the corresponding fitting curve (P = U^2^).

The relationship between the voltage values and power is depicted in Figure 11. The fitting curve demonstrates that actual power is proportional to the square of the measured voltage. Subsequent calculations of power can be derived from the voltage values collected by the ADC. This enables the computation of the standing wave ratio based on the incident and reflected power.

### 3.4. Acoustic Testing and Ultrasound Thermal Ablation Experiment

**Figure 12:**
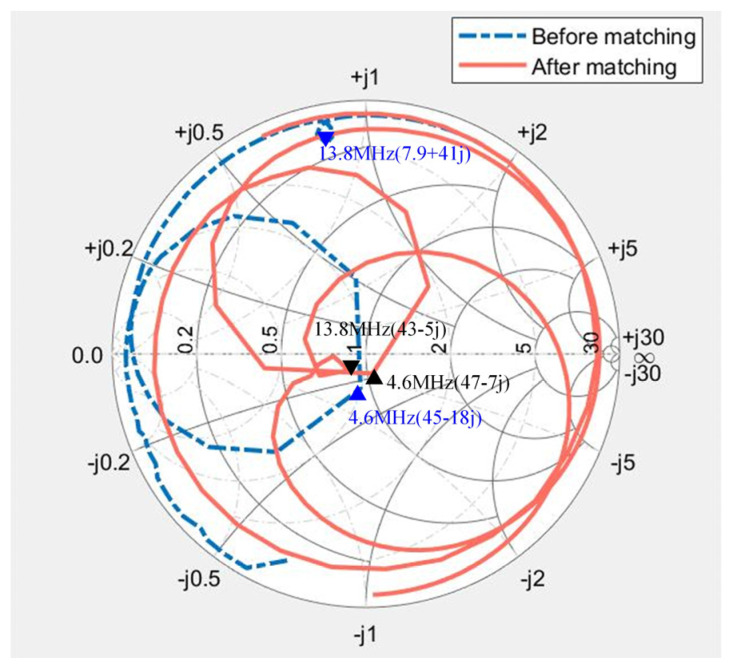
Frequency response of the transducer with and without the DFEIM circuit.

**Figure 13:**
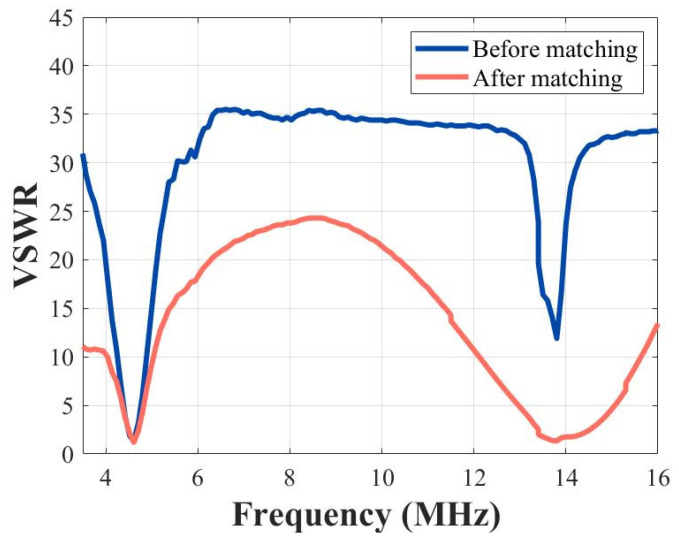
Standing wave ratio comparison for the transducer with and without the DFEIM circuit.

As depicted in Figure 12, the impedance values of the transducer without DFEM circuit at the fundamental frequency and third harmonic were 45 − 18j and 7.9 + 41j, respectively. With DFEM circuit, these values changed to 47 − 7j and 43 − 5j for the fundamental frequency and third harmonic, respectively. The matched transducer closely approximated the target matching point of 50 ohms and 0 phase at both frequency points. The voltage standing wave ratio (VSWR) before and after matching is illustrated in Figure 13. The matching circuit reduced the standing wave ratio at the fundamental frequency from 1.3 to 1.2, and, at harmonics, it decreased from 11.9 to 1.3.

The acoustic power output of the transducer is presented in Table 2. When subjected to 18 W of electrical power individually applied to two frequencies, without the DFEIM circuit, the acoustic power output at the fundamental frequency and the third harmonic was 11.1 W and 2.6 W, respectively. With the DFEIM circuit, the acoustic power output for the fundamental frequency and the third harmonic experienced an increase, reaching 11.9 W and 6.3 W, respectively.

**Figure 14:**
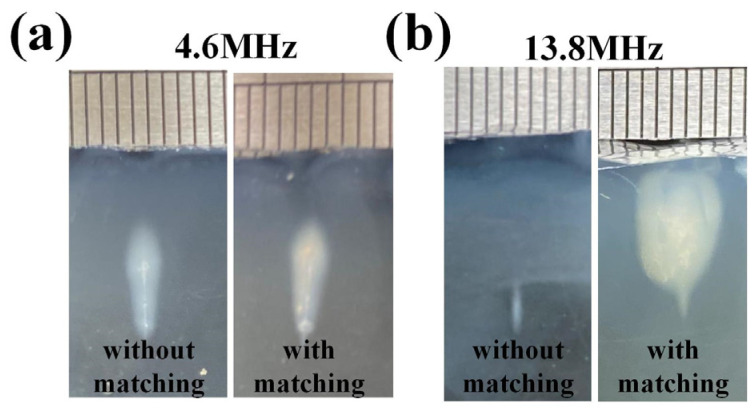
Side view of the thermal damage area in the phantom, comparing scenarios with and without the DFEIM circuit, at both (**a**) 4.6 MHz and (**b**) 13.8 MHz.

Under 18 W input electrical power, the thermal damage area within the phantom is depicted in Figure 14. The comparison involves the fundamental frequency and third harmonic excitations without the DFEIM circuit and with the DFEIM circuit. The implementation of the DFEIM circuit resulted in a 7% increase in the fundamental frequency ablation area (14 mm² versus 15 mm²) and a significant 21.7-fold amplification in the harmonic ablation area (3 mm² versus 65 mm²).

**Figure 15:**
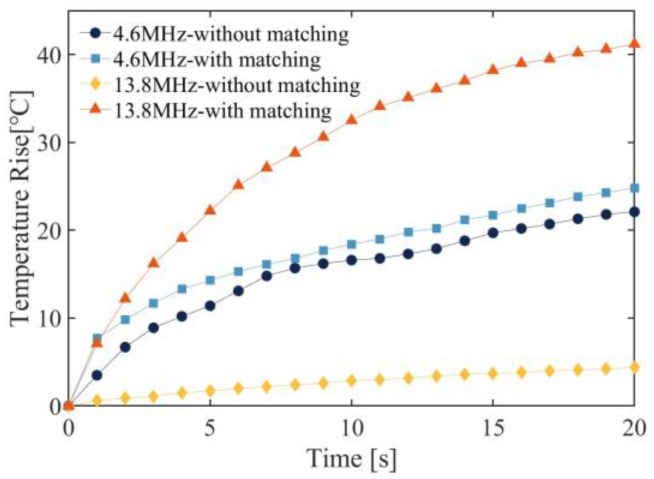
Temperature rise curve at the focal point of the phantom, comparing scenarios with and without the DFEIM circuit.

Figure 15 illustrates the temperature of the focal area of the transducer under fundamental frequency and third harmonic excitation before and after matching. The DFEIM circuit elevated the temperature rise at the fundamental frequency focus from 22 °C to 24.8 °C and increased the temperature rise at the harmonic focus from 4.4 °C to 41.2 °C.

## 4. Discussion

Most traditional EIM technologies are performed for a single frequency, and conventional dual-frequency transducers typically need two distinct matching circuits for two frequencies. When a transducer is tasked with operating at multiple frequencies, the EIM network must be replaced [15]. This approach limits the simultaneous transmission of two frequencies or the rapid switching between them. The DFEIM network topology proposed in this article addresses two frequency points concurrently, enabling the simultaneous transmission of two frequencies or swift switching between them. Diverging from traditional single-frequency EIM, the DFEIM network is more intricate, involving a greater number of components.

The conventional approach to determine component parameter values in matching involves formula calculations or the enumeration of various parameter combinations to identify optimal solutions for obtaining capacitance and inductance values. However, DFEIM poses challenges due to increased frequency points and the consideration of numerous components. Enumerating various combinations of parameter values becomes unwieldy, necessitating an extensive number of enumerations, thereby constraining efficiency. As an optimization algorithm [36], the genetic algorithm excels in discerning optimal solutions amid intricate and diverse conditions [37]. Consequently, this article employs the genetic algorithm to efficiently and swiftly identify optimal parameter combinations, innovatively applying it to the parameter optimization of DFEIM technology.

The DFEIM system presented in our work features adjustable parameters covering a broad range, making it applicable to most ultrasound therapy transducers. The inclusion of a feedback circuit facilitates monitoring the suitability of the matching circuit, allowing for re-matching through an optimization algorithm. This substantially enhances the flexibility of matching and ensures the safety of the ultrasound treatment system.

In intricate networks, the evaluation function often exhibits a high-order nonlinear relationship with component values, and the addition of components can diminish the efficacy of the search process. Existing EIM optimization algorithms can be categorized into two groups: probabilistic optimization algorithms and deterministic optimization algorithms. Deterministic optimization algorithms often converge to local optimal solutions, yielding suboptimal processing results. In contrast, probabilistic optimization algorithms introduce randomness to circumvent the issue of local extreme values. Three prominent probabilistic optimization algorithms include the annealing algorithm [38], particle swarm algorithm [39], and genetic algorithm [23,40]. As an evolutionary algorithm, the genetic algorithm demonstrates heightened robustness and applicability, efficiently addressing the complexities of DFEIM scenarios. Consequently, the genetic algorithm is chosen as the optimization algorithm in this article.

The simulation of the DFEIM circuit was validated on a Smith chart. The results, as illustrated in Figure 10, demonstrate that the DFEIM circuit computed through this method matched the impedance values at the two frequency points with the target impedance value. The results from transducer testing also corroborate this observation (Figure 12). This DFEIM matched the impedance values of the transducer at the two frequency points concurrently with the target impedance, reducing the standing wave ratio simultaneously (Figure 13) and increasing the output power by 7.2% and 142%, respectively (Table 2).

The results of the thermal ablation tests suggest that the DFEIM circuit has a noticeable impact on the ablation areas at both the fundamental and harmonic frequencies (Figure 14 and Figure 15). The increase in the fundamental frequency ablation area is relatively modest (7%), while there is a substantial enhancement in the harmonic frequency ablation area (21.7-fold increase). This information is valuable for assessing the effectiveness of the DFEIM circuit in influencing thermal ablation outcomes in the phantom body under the specified experimental conditions. Furthermore, the presence of the DFEIM circuit results in the ablation zone within the phantom being closer to the transducer compared to its absence. This phenomenon primarily stems from cavitation and boiling, leading to the formation of tadpole-like lesions migrating towards the transducer [41]. The escalation in peak pressure accelerates heating, fostering quicker damage formation, while larger bubbles generated by boiling exacerbate the distortion of the lesion shape. This distortion, resembling tadpoles, arises from the reflection of vapor bubbles. Although the sound power at 13.8 MHz (6.3 W) is lower than that at 4.6 MHz (11.9 W), the sound absorption coefficient at 13.8 MHz in the phantom is approximately 3.3 times higher than the sound absorption coefficient at 4.6 MHz, resulting in a larger thermal damage area generated by 13.8 MHz.

Dual-frequency ultrasound thermal ablation systems often excel in delivering heat more rapidly, resulting in abbreviated treatment durations. This proves advantageous for enhancing surgical efficiency, minimizing patient discomfort, and mitigating risks during surgery. The versatility of dual-frequency technology allows for the adjustment of frequencies to suit different types of tissues. Certain tissues may exhibit increased sensitivity to specific frequencies, enabling the customization and optimization of treatment effects through the use of two frequencies. Simultaneous application of dual-frequency technology involves two ultrasound waves with distinct frequencies, enabling precise control of heat energy transfer. This facilitates more targeted thermal ablation within the designated area, thereby minimizing damage to surrounding normal tissue. DFEIM concurrently enhances the transmission efficiency of the ultrasound system and transducer at two frequencies, contributing to the overall stability of the equipment. In applications such as ultrasound thermal ablation and ultrasound thrombolysis, dual-frequency ultrasound demonstrates superior treatment efficiency compared to single frequency. With the growing popularity of dual-frequency ultrasound treatment, the prospects for DFEIM appear promising.

While this method exhibits a degree of universal adaptability, practical implementation may encounter discrepancies between simulation-calculated parameter values and actual deployment due to inherent errors and gradients in capacitor and inductor values. In the dual-frequency DFEIM network topology, the scope has been limited to expanding the simplest L-shaped network in a single frequency into a dual-frequency topology. Other common single-frequency types, such as T-type, Π-type, and broadband networks, have not been explored in this research. The feedback system in this system serves solely as a reference for evaluating the effectiveness of the proposed method and does not actively participate in the optimization algorithm. The choice of the genetic algorithm for optimization is based on experience and lacks a comparative analysis with other algorithms.

In future work, considerations will include incorporating the power and standing wave ratio of the feedback circuit into the optimization algorithm for real-time adaptive matching. Exploration of multiple optimization algorithms will be undertaken to study faster and more accurate dual-frequency matching optimization algorithms. Furthermore, the investigation will extend to exploring dual-frequency matching networks with more diverse topological structures, optimizing the overall topology of DFEIM. In this study, we primarily introduce the design of the DFEIM circuit. In future investigations, we intend to carry out additional finite element simulations and design experiments using phantoms and animal models to evaluate the dose-effect relationship of dual-frequency HIFU.

Despite certain limitations, the design method proposed in this article effectively addresses the challenges associated with matching dual-frequency power ultrasound transducers and power amplifier systems. It simultaneously enhances the transmission efficiency and acoustic power output at two frequency points, leading to improved thermal ablation efficiency at two frequencies. This method holds promising application prospects in the realm of dual-frequency ultrasound treatment.

## 5. Conclusions

This paper presents a design methodology for a DFEIM network tailored for ultrasound transducers. The approach outlined herein involves selecting a DFEIM network topology based on the frequency response of the transducer to be matched, followed by computing component values using a genetic algorithm. This method notably streamlines the implementation of intricate DFEIM configurations, thereby enhancing matching efficacy. The simulation outcomes illustrate that the topology and component values derived by the DFEIM network, employing the genetic algorithm, effectively match the two frequencies of the transducer with the target impedance values. Subsequently, the practical deployment and testing of the transducer’s actual output through the DFEIM network were executed. Experimental findings demonstrate that the DFEIM network enhances the acoustic power output of the transducer across both frequencies. In the thermal ablation experiment conducted on an in vitro phantom, the temperature rise rate and thermal damage area of the ultrasound transducer at two frequency points were augmented through the DFEIM network. The proposed methodology holds promising prospects for applications in dual-frequency ultrasound treatment.

## Figures and Tables

**Table 1:** Parameter values of component obtained by genetic algorithm.

Component	C1 (nF)	L1 (nH)	C2 (pF)	L2 (nH)
Values	2.84	2344	450	958

**Table 2:** Acoustic power measurements of the transducer without and with DFEIM circuit under 18W electrical power.

Frequency	Input Electrical Power (W)	Output Acoustic Power (W)	
		Without DFEIM	With DFEIM
4.6 MHz	18	11.1	11.9
13.8 MHz	18	2.6	6.3

## Data Availability

Data are contained within the article.
